# Concurrent therapy-related acute myeloid leukemia and lymph node tuberculosis following treatment for lung squamous cell carcinoma: a case report and literature review

**DOI:** 10.3389/fonc.2026.1820207

**Published:** 2026-04-15

**Authors:** Yonglin Yu, Dongmei Yang, Xiaoju Chen, Yong Chen

**Affiliations:** 1 Department of Nursing, Affiliated Hospital of North Sichuan Medical College, Nanchong, Sichuan, China; 2 Department of Respiratory and Critical Care Medicine, The Affiliated Hospital of North Sichuan Medical College, Nanchong, China; 3 Department of Respiratory and Critical Care Medicine, Sichuan Friendship Hospital, Chengdu, China; 4 The First Clinical Medical College, General Hospital of Ningxia Medical University, Ningxia, China

**Keywords:** case report, lung squamous cell carcinoma, lymph node tuberculosis, therapy-related acute myeloid leukemia, therapy-related complications

## Abstract

Concurrent development of therapy−related acute myeloid leukemia (t−AML) and lymph node tuberculosis (LNTB) following comprehensive anti−tumor therapy for locally advanced lung squamous cell carcinoma (LSCC) is extremely rare in clinical practice. This is not a new biological concept but represents a rarely documented clinical scenario in the setting of neoadjuvant chemoimmunotherapy, surgery, and anti–PD−1 maintenance therapy. Herein, we systematically summarize the clinical features, pathogenesis and individualized therapeutic strategies of these two concurrent rare complications based on a single rare case and the latest relevant literature, to provide a reference for clinical diagnosis and treatment. A 54-year-old male patient was initially diagnosed with locally advanced LSCC. After four cycles of neoadjuvant therapy with carboplatin, albumin-bound paclitaxel and pembrolizumab, the tumor lesion regressed markedly. Thoracoscopic right upper lobectomy was then performed, followed by maintenance immunotherapy with single-agent pembrolizumab postoperatively. Four months after maintenance therapy, the patient developed abnormalities on routine blood work, low-grade fever, fatigue and superficial lymphadenopathy. t-AML was confirmed by bone marrow aspiration, immunophenotyping, gene mutation and cytogenetic examinations, accompanied by breast cancer susceptibility gene 2 (*BRCA2*), DNA (cytosine-5)-methyltransferase 3 alpha (*DNMT3A*), and isocitrate dehydrogenase 2 (*IDH2*) mutations, and positivity for lysine (K)-specific methyltransferase 2A partial tandem duplication (*KMT2A-PTD*). Meanwhile, LNTB was diagnosed by lymph node aspiration pathology combined with tuberculosis-specific assays. The patient was treated with an optimized quadruple anti-tuberculosis regimen (HZEM), and induction chemotherapy for AML with VA regimen (venetoclax plus azacitidine) plus revumenib, and supportive therapy. Subsequently, the patient achieved partial remission of leukemia, with no uncontrollable severe adverse events. In this case, LSCC was managed with neoadjuvant therapy, thoracoscopic right upper lobectomy and postoperative maintenance therapy with single agent pembrolizumab. The development of t−AML is primarily driven by cytotoxic DNA damage induced by chemotherapeutic agents, whereas the potential contribution of immune checkpoint inhibitors remains largely speculative. The potential contribution of immune checkpoint inhibitors via immune microenvironmental disturbance remains largely speculative and insufficiently documented by current clinical evidence. The impaired immune function caused by comprehensive anti-tumor therapy may further elevate the risk of LNTB. The overlapping clinical manifestations of the two concurrent diseases substantially increase diagnostic difficulty. Timely and thorough bone marrow examination, lymph node pathological biopsy and tuberculosis-specific screening are the keys to early and accurate diagnosis.

## 1 Introduction

Lung squamous cell carcinoma (LSCC) is one of the most common histological subtypes of lung cancer, accounting for 20–30% of all pulmonary malignancies and carrying an extremely high mortality rate (1).Unfortunately, most patients are diagnosed at an advanced stage and are not eligible for upfront radical surgical resection (2). In recent years, immune checkpoint inhibitor (ICI) combination chemotherapy has become the first-line treatment for locally advanced LSCC. This approach can significantly reduce tumor burden, improve resection rates and short-term clinical outcomes, but may also increase the risk of long-term complications (3, 4). Therapy−related acute myeloid leukemia (t−AML) is a well−recognized complication caused mainly by cytotoxic chemotherapy and radiotherapy, while the role of immunotherapy remains unclear (5). Studies have shown that AML arising following chemotherapy and/or radiotherapy for previous neoplastic or non-neoplastic diseases accounts for 10% of all adult AML cases (6). Compared with *de novo* AML, t-AML is frequently accompanied by adverse cytogenetic abnormalities and tumor protein p53(*TP53*)mutations, conferring a dismal prognosis with a median survival of 8–14 months after chemotherapy (7).Allogeneic hematopoietic stem cell transplantation (allo-HSCT) may provide a curative opportunity, but the overall survival remains limited (8). Extrapulmonary tuberculosis accounts for 10–25% of all tuberculosis cases (9), and lymph node tuberculosis (LNTB) is the most common form of extrapulmonary tuberculosis (10), predominantly involving the cervical region, followed by the axillary and inguinal regions (11). Pathologically, it is characterized by granulomatous lesions and caseous necrosis caused by Mycobacterium tuberculosis (MTB) proliferation in lymphoid tissues, and clinically manifests as painless lymphadenopathy, low-grade fever, night sweats and fatigue. The lung cancer microenvironment, characterized by chronic inflammation, hypoxia and abnormal vascular networks, facilitates MTB proliferation, dissemination and reactivation (12, 13). During ICI treatment, PD-1 blockade induces immune reconstitution inflammatory syndrome (14, 15), which not only enhances CD4+ T-cell responses against MTB but also disrupts granulomas via excessive Th1 activation, increasing the risk of MTB dissemination (16). Conventional chemotherapy similarly elevates the risk of MTB infection or reactivation; the resulting myelosuppression, neutropenia, mucosal barrier damage, and platinum-induced impairment of CD4+ T-cell function collectively weaken host anti-tuberculosis immunity (17, 18). The prevalence of tuberculosis is significantly higher in patients with hematologic malignancies than in the general population, especially among those with leukemia (19). The high incidence of tuberculosis in leukemia patients is associated with reduced CD4+ T-cell counts, impaired CD8+ T-cell function, a decreased CD4+/CD8+ ratio, and systemic immunosuppression caused by the underlying disease, chemotherapy or hematopoietic stem cell transplantation, all of which contribute to the reactivation of latent tuberculosis tuberculosis. Notably, the prevalence is markedly higher in patients with acute myeloid leukemia than in those with other hematologic malignancies (20).

To date, no prior case has reported concurrent t−AML and LNTB in LSCC patients treated with neoadjuvant chemoimmunotherapy, surgical resection, and maintenance ICI therapy. This rare combination presents overlapping manifestations and complex therapeutic challenges. The treatment regimen is highly complex, requiring simultaneous anti-tuberculosis and anti-leukemic therapy, avoidance of drug-drug interactions, and a balance between therapeutic efficacy and adverse reactions.

## 2 Case report

### 2.1 General information

A 54-year-old male patient had a 30-year smoking history (20 cigarettes/day) and had never quit smoking. He had no history of tuberculosis, familial hematological diseases, autoimmune diseases, or other chronic medical conditions.

In March 2025, he presented to the Department of Respiratory Medicine of our hospital due to hemoptysis lasting more than 4 days, with no hot flashes, night sweats, chest pain, dyspnea, palpitations, or other accompanying symptoms. No relevant past medical history was noted.

#### 2.1.1 Physical examination

Body temperature was 36.8°C. The patient was conscious and in good general condition. No enlarged superficial lymph nodes were palpable. Bilateral breath sounds were clear, with no dry or moist rales. Heart rate was 78 beats/min with a regular rhythm, and no pathological murmurss were detected in any valve auscultation area. The abdomen was soft and flat, with no tenderness or rebound tenderness; no hepatosplenomegaly was observed, and no edema was noted in both lower extremities.

#### 2.1.2 Auxiliary examinations

##### 2.1.2.1 Whole-body PET-CT revealed

**Figure 1:**
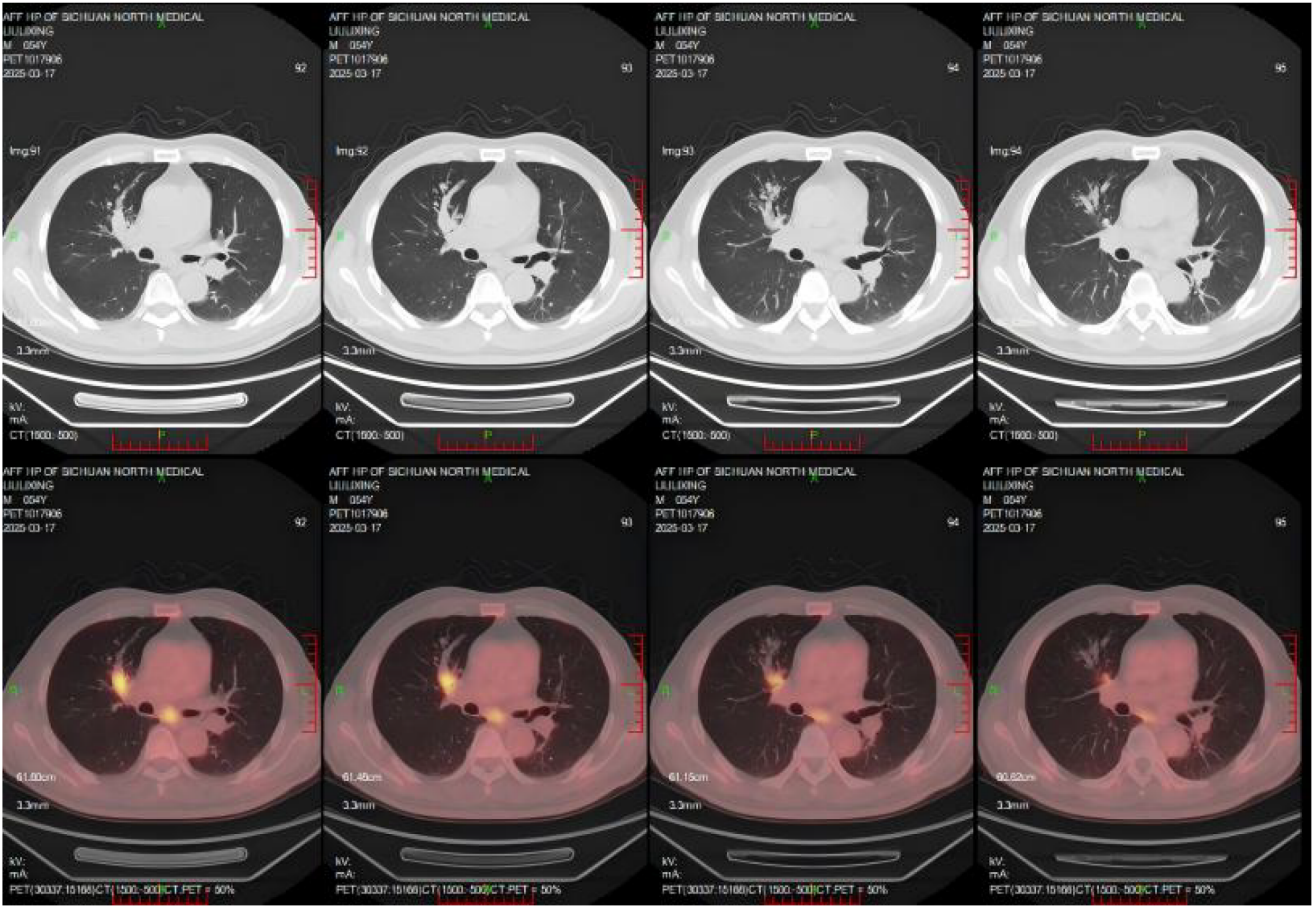
PET-CT findings demonstrated bronchial truncation in the anterior segment of the right upper lobe, local patchy-nodular soft-tissue density with increased density in the distal lung field, mild bronchiectasis in some bronchi, and abnormally elevated FDG metabolism. Enlarged lymph nodes with abnormally elevated FDG metabolism were also observed in the pretracheal, retrocaval, and subcarinal regions.

Bronchial truncation in the anterior segment of the right upper lobe, local patchy-nodular soft-tissue density with increased density in the distal lung field, mild bronchiectasis in some bronchi, and abnormally elevated FDG metabolism. Enlarged lymph nodes in the pretracheal, retrocaval, and subcarinal regions with abnormally elevated FDG metabolism (**Figure 1**).

##### 2.1.2.2 Combined with clinical history, the findings were interpreted as follows

**Figure 2:**
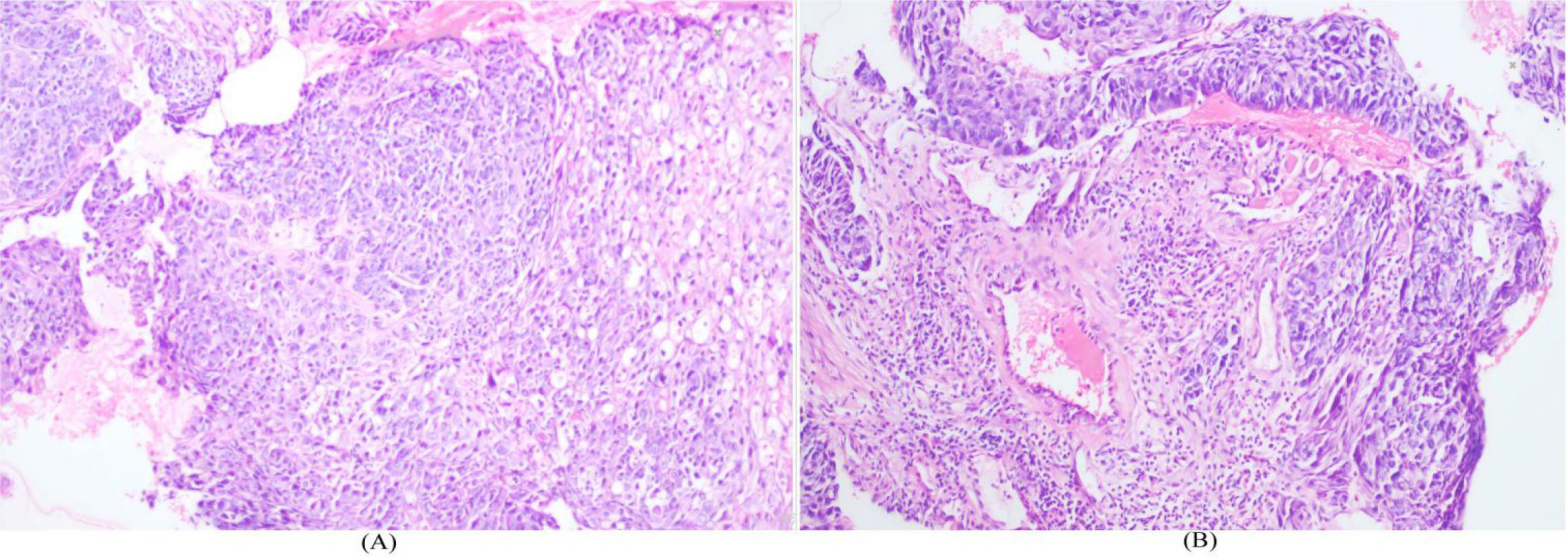
**(A, B)** Histopathological examination via fiberoptic bronchoscopy demonstrated squamous cell carcinoma.

(1) Findings consistent with carcinoma of the anterior segment of the right upper lobe, accompanied by obstructive pneumonia and mild bronchiectasis in the distal lung field; (2) Possible metastasis to the aforementioned lymph nodes; (3) No abnormally elevated FDG metabolism in other regions. Pathological biopsy via fiberoptic bronchoscopy confirmed squamous cell carcinoma (**Figure 2**). Routine blood test, liver and renal function, electrolytes, and tuberculosis-related examinations (TST, IGRA) showed no significant abnormalities. Based on the above investigations, the patient was diagnosed with squamous cell carcinoma of the right upper lobe (cT2N2M0, stage IIIA).

### 2.2 Initial treatment (multimodality therapy for squamous cell carcinoma of the right upper lung)

According to the patient’s age, clinical condition and imaging findings, an individualized comprehensive treatment strategy was established following discussion by a multidisciplinary team (MDT) including thoracic surgery, respiratory medicine and oncology. The detailed therapeutic process is as follows:

#### 2.2.1 Neoadjuvant therapy

The patient received a combined regimen of carboplatin (400 mg/m^2^, intravenous infusion, day 1) plus albumin−bound paclitaxel (260 mg/m^2^, intravenous infusion, day 1) and pembrolizumab (2 mg/kg, intravenous infusion, day 2). The treatment cycle was 21 days, and a total of four cycles were administered.

#### 2.2.2 Surgical treatment

**Figure 3:**
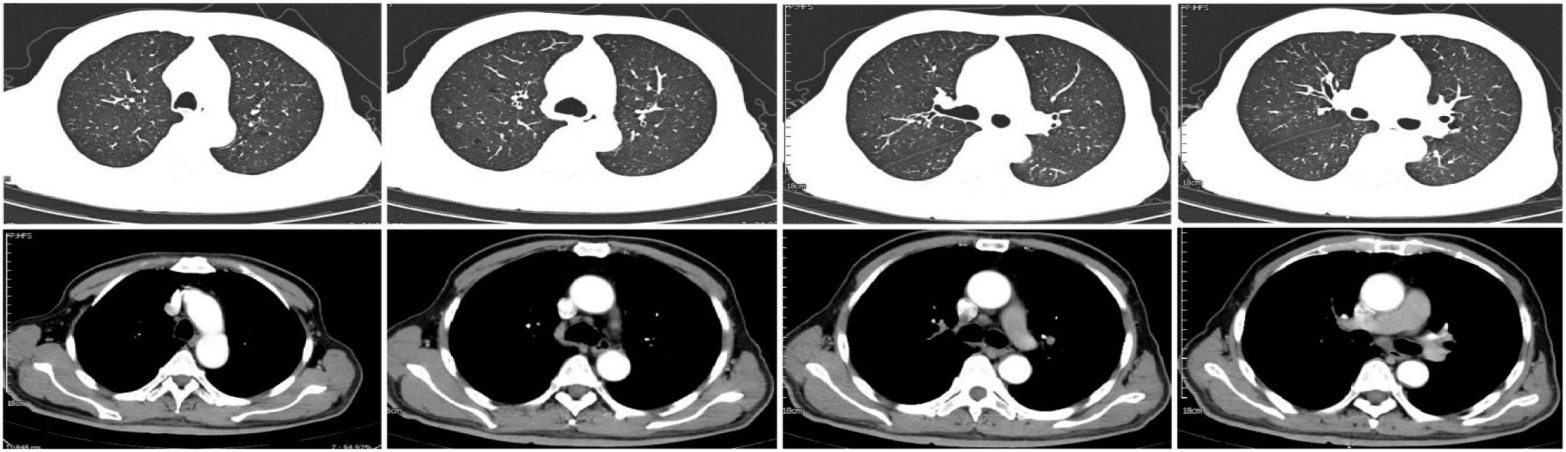
After 4 cycles of neoadjuvant therapy, follow-up contrast-enhanced chest CT showed that following neoadjuvant treatment for squamous cell carcinoma of the right upper lobe, the primary lesion was significantly reduced in size, and obstructive pneumonia was almost completely resolved.

After completion of 4 neoadjuvant cycles, follow−up chest CT showed that the primary tumor of squamous cell carcinoma in the right upper lobe was markedly reduced in size, and obstructive pneumonia was almost completely resolved (**Figure 3**). The patient was evaluated as achieving a partial response (PR) with no contraindications to surgery. Video−assisted thoracoscopic right upper lobectomy was performed in July 2025. The operation was uneventful. Postoperative pathology confirmed squamous cell carcinoma of the right upper lobe with negative surgical margins, and no metastatic carcinoma was detected in hilar or mediastinal lymph nodes. The postoperative pathological stage was squamous cell carcinoma of the right upper lobe (ypT0N0M0, Stage I).

#### 2.2.3 Maintenance therapy

**Figure 4:**
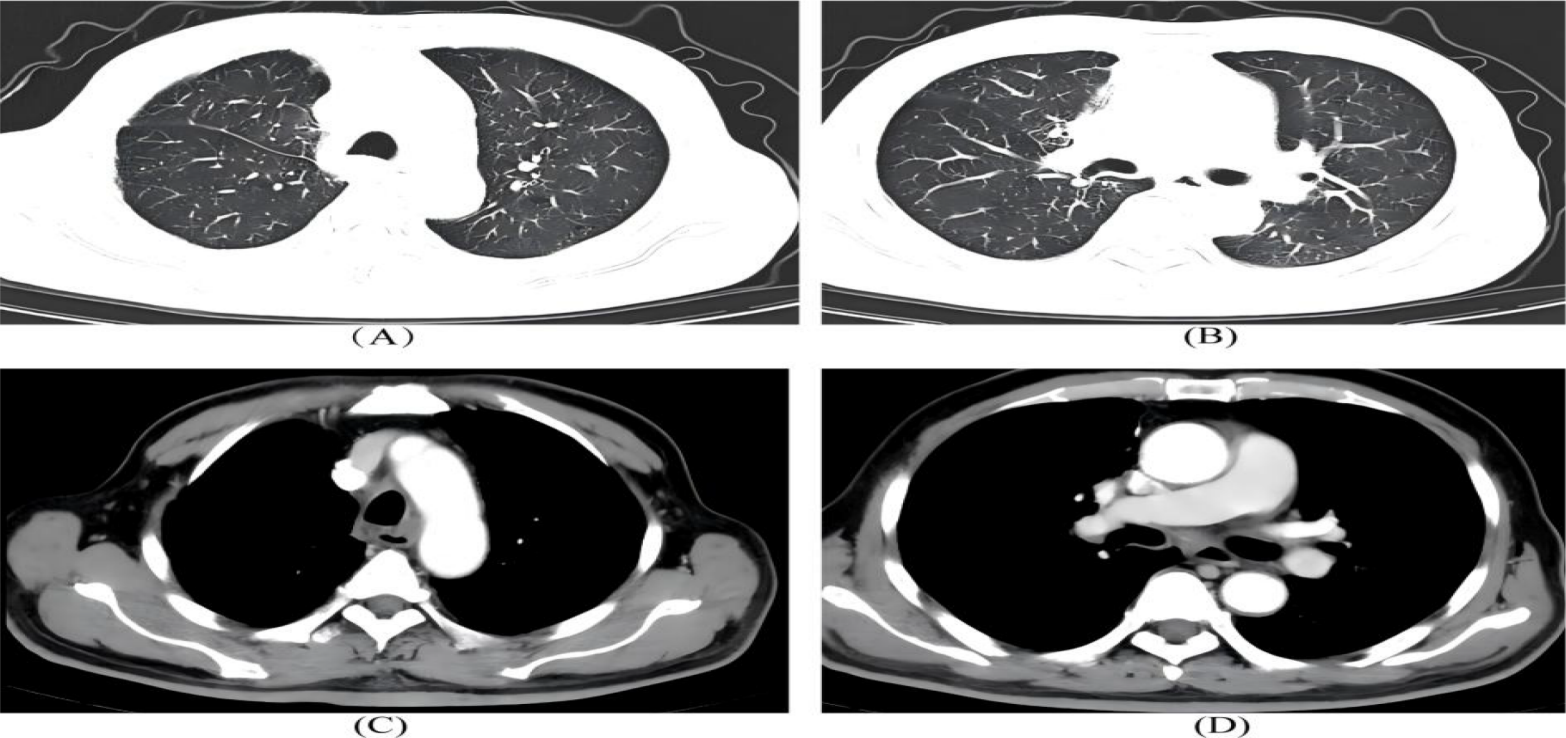
**(A–D)** Status post video−assisted thoracoscopic right upper lobectomy.Strip−like high−density shadows and a small amount of band−like increased opacity are visible in the surgical bed.Cord−like high−density shadows are present in the right residual lung, with no abnormal mass lesions identified.

The patient recovered smoothly postoperatively without surgery-related complications. Maintenance therapy with pembrolizumab monotherapy was initiated in August 2025 (same dosage as before, 1 cycle every 21 days) for a planned duration of 1 year. Regular follow-up examinations were performed during maintenance therapy: routine blood tests and liver/renal function tests every 2 months, and chest CT every 3 months. Follow-up chest CT in December 2025 is presented in (**Figure 4**).

### 2.3 Clinical manifestations and diagnosis of secondary diseases

In December 2025, four months after the initiation of pembrolizumab monotherapy for maintenance, the patient was readmitted presenting with persistent low-grade fever (body temperature 37.5–38.0 °C), aggravated fatigue, and enlarged cervical and axillary lymph nodes for 3 days. Pembrolizumab was discontinued, and comprehensive examinations were performed to establish a definitive diagnosis.

#### 2.3.1 Hematological abnormalities and diagnosis of therapy-related acute myeloid leukemia

##### 2.3.1.1 Initial routine blood test on admission showed

white blood cell count 6.7×10^9^/L, red blood cell count 2.62×10^12^/L, hemoglobin 85 g/L, platelet count 57×10^9^/L, absolute neutrophil count 0.16×10^9^/L, neutrophil percentage 2.40%.The results indicated severe neutropenia and dysplasia. Further examinations were performed, including bone marrow aspiration, immunophenotyping, cytogenetics, and next-generation sequencing (NGS).

**Figure 5:**
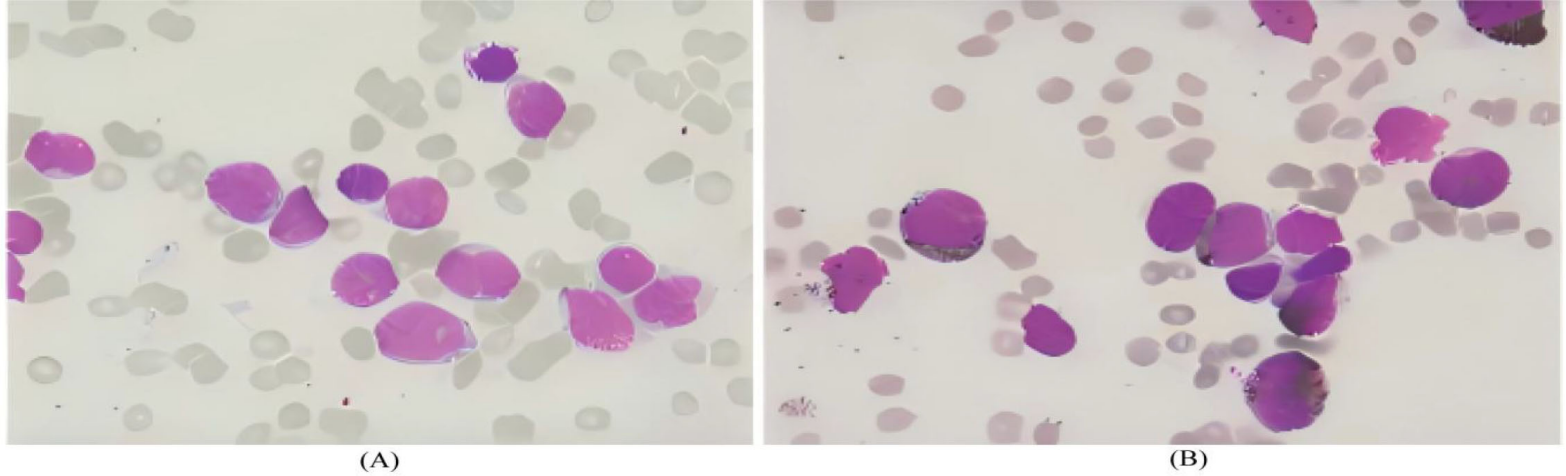
**(A, B)** Bone marrow findings: Adequate aspiration, good smearing, and excellent staining, with particulates (+) and oil droplets (+). Hypoactive cellularity (G = 53%, E = 17%, G/E=4.29:1). Granulocytic series proportion was normal, but myeloblasts increased to 80%. Erythroid series was normal, predominantly intermediate and late erythroblasts, with mature red blood cell morphology generally normal. Lymphoid series was normal (mature lymphocytes). Thirty-two megakaryocytes were observed in the entire smear, with scattered platelets.

**Figure 6:**
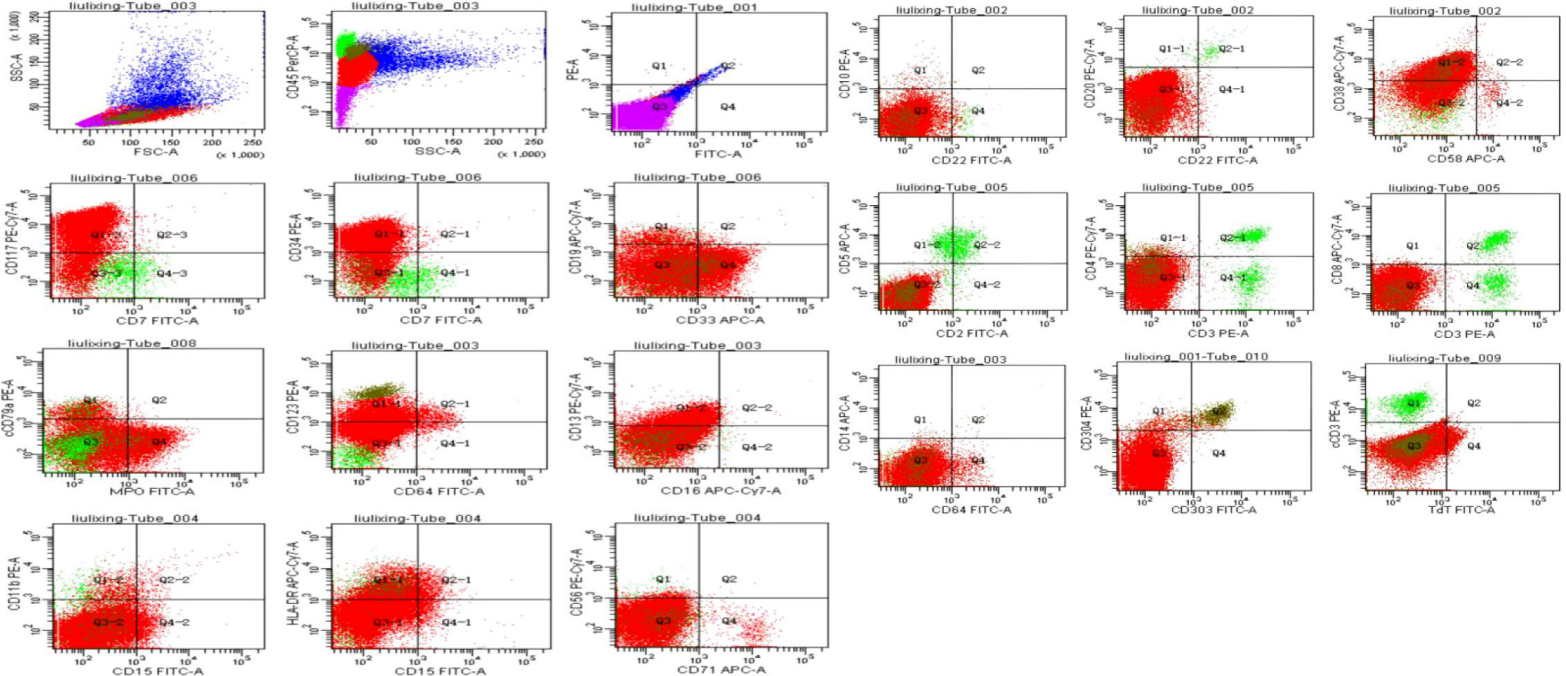
Gating analysis was performed on the CD45/SSC dot plot. An abnormal cell population was detected in the blast region, accounting for 76.5% of nucleated cells, which mainly expressed HLA-DR, CD13, CD33, CD34, CD38, CD117, CD123 and MPO.The myeloid region comprised 13% of nucleated cells, including 10.7% of nucleated cells representing myeloid cells.In addition, CD123bright-positive cells (olive green) accounted for 2.3% of nucleated cells, expressing HLA-DR, CD4, CD33, CD38, CD303 and CD304, consistent with a dendritic cell origin.Lymphoid proliferation was significantly suppressed.The relative proportions were as follows: lymphocytes (green) 4.5%, abnormal cells (red) 76.5%, myeloid cell region (blue) 13%, and nucleated erythrocyte region (purple) 6%.

**Figure 7:**
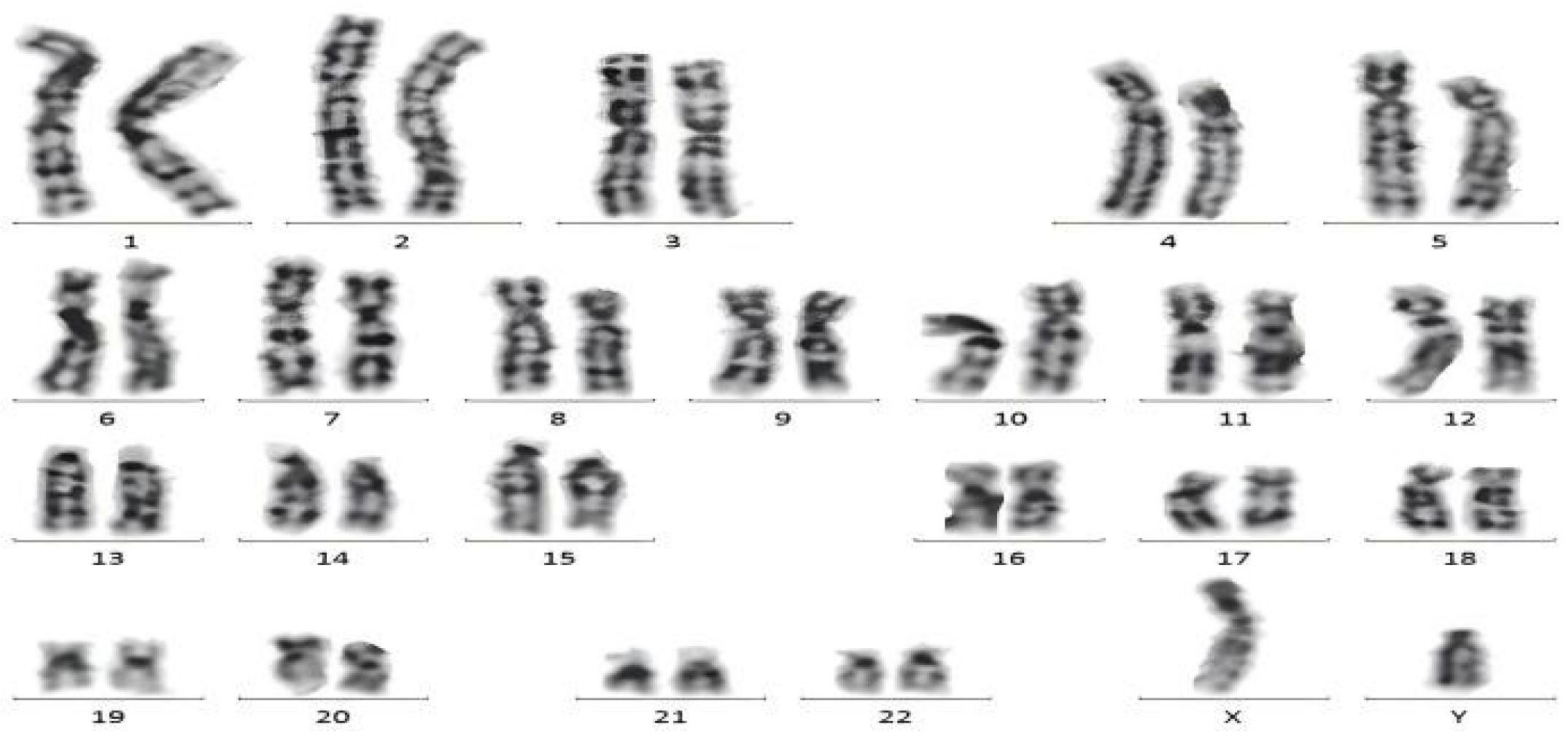
Metaphase analysis was performed on the cultured specimen, and no clonal chromosomal abnormalities were detected.

**Figure 8:**
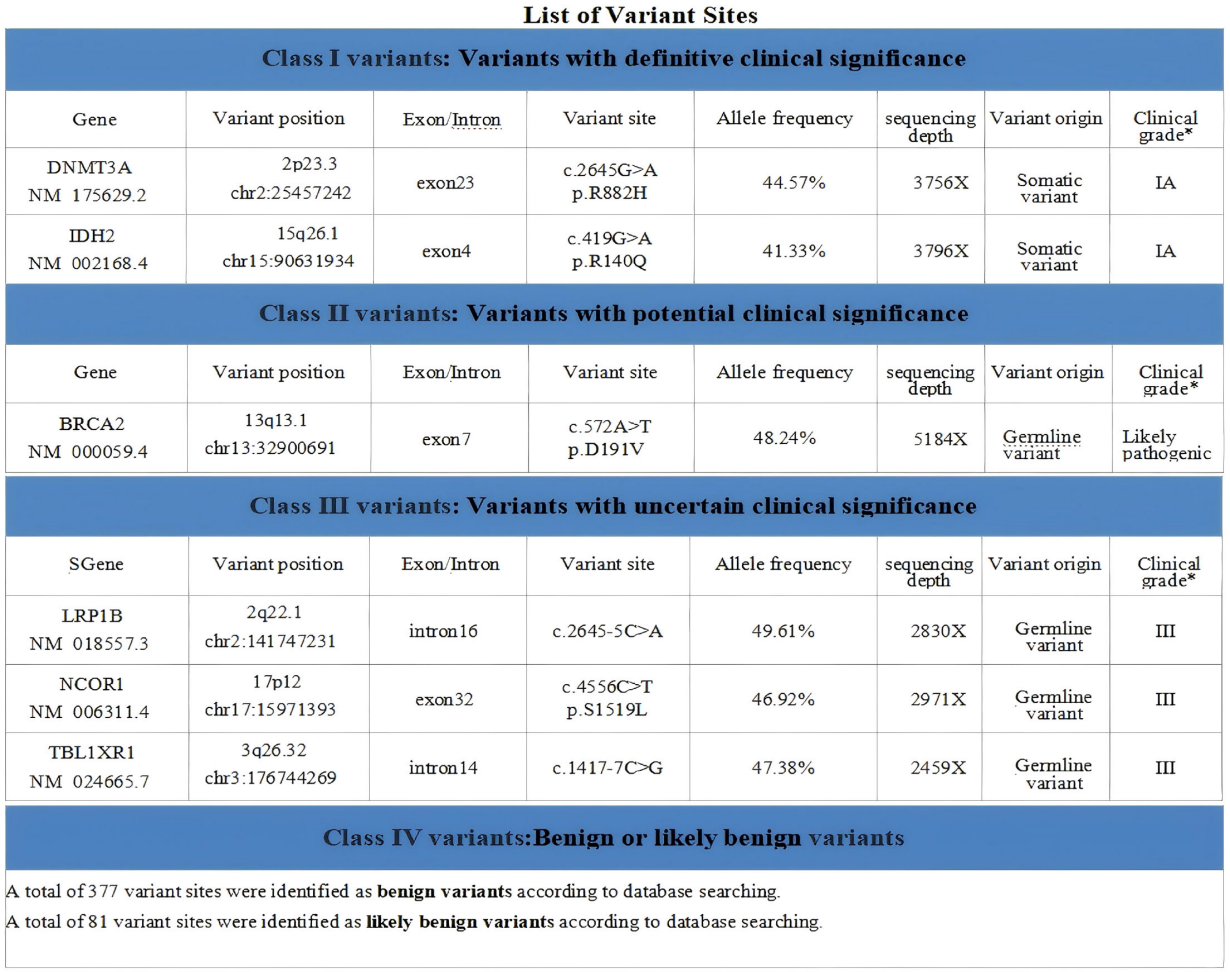
Four categories of gene variants were identified in this sample, namely Class I variants (variants with definitive clinical significance), Class II variants (variants with potential clinical significance), Class III variants (variants with uncertain clinical significance), and Class IV variants (benign or likely benign variants).

1. Bone marrow morphology supported a diagnosis of acute myeloid leukemia (**Figure 5**).
2. Immunophenotyping demonstrated AML with elevated plasmacytoid dendritic cells (pDCs) (**Figure 6**).
3. Bone marrow chromosome analysis: 46, XY; no clonal cytogenetic abnormalities were detected (**Figure 7**).
4. Myeloid hematologic neoplasm gene testing revealed mutations in Breast Cancer Susceptibility Gene 2(*BRCA2*) (variant allele frequency 48.24%), DNA (cytosine-5-)-methyltransferase 3 alpha (*DNMT3A*) (44.57%), and Isocitrate Dehydrogenase 2(*IDH2*) (41.33%), was positive for the Partial Tandem Duplication of Lysine (K)-Specific Methyltransferase 2A(*KMT2A−PTD*) fusion gene, and was negative for FMS-like tyrosine kinase 3-internal tandem duplication (*FLT3−ITD*) (**Figure 8**). NGS was performed on the DNBSEQ−T1+ platform. Sequencing coverage was verified to ensure full capture of all targeted gene regions. Variants were detected and annotated using a validated bioinformatics pipeline, then interpreted by experienced hematologists using a laboratory−specific hematologic neoplasm database to generate the final clinical report.

#### 2.3.2 Lymphadenopathy and diagnosis of LNTB

**Figure 9:**
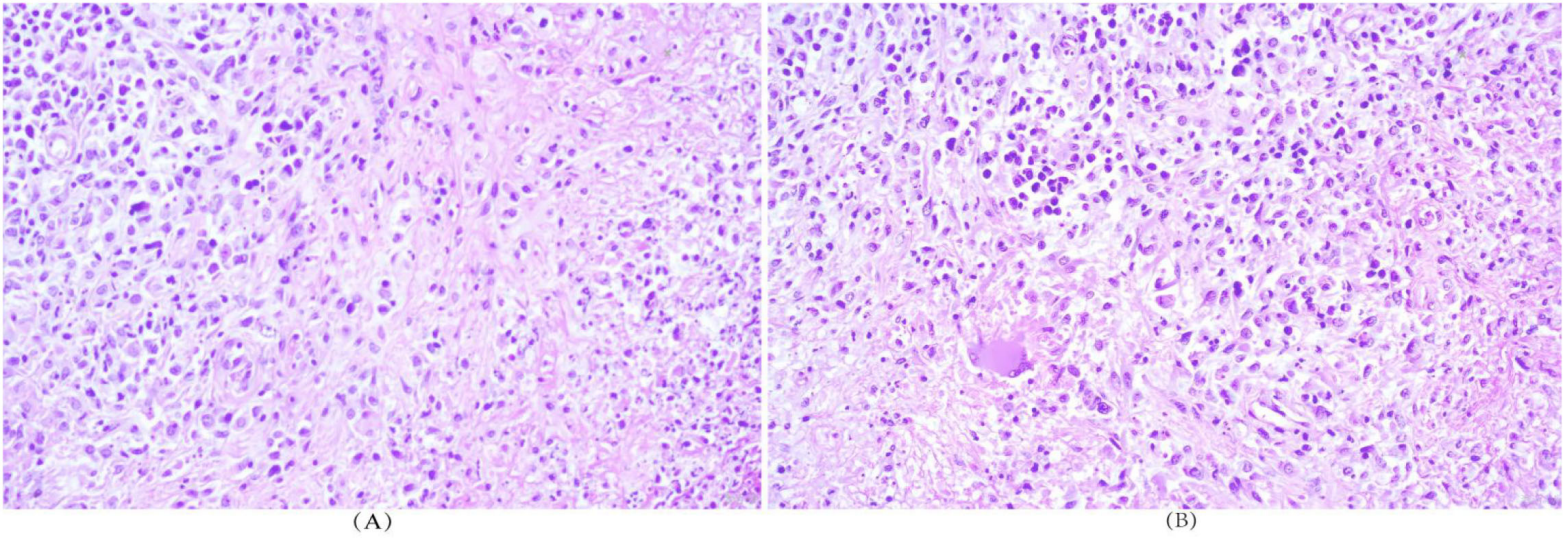
**(A, B)** Granulomatous inflammation with necrosis; morphological features are consistent with tuberculous lesions.

Physical examination revealed painless enlarged lymph nodes in the right neck, the largest measuring approximately 2.6 cm×2.1 cm, with moderate consistency, satisfactory mobility and no tenderness. Cervical lymph node ultrasonography showed multiple enlarged lymph nodes in the neck and axillae, with unclear corticomedullary demarcation, heterogeneous internal echo and a small number of liquefactive foci in some lesions. Ultrasound−guided needle biopsy of the cervical lymph node was performed, and pathological examination demonstrated granulomatous inflammation with typical caseous necrosis (**Figure 9**). Further tuberculosis−related examinations were completed: the tuberculin skin(TST) test showed an induration diameter of 13 mm (positive), TB−IGRA was positive, tuberculosis antibody was positive, ESR was 85 mm/h, and CRP was 122 mg/L, all of which supported a diagnosis of tuberculosis infection. Combined with the patient’s clinical manifestations, pathological results and tuberculosis−related examinations, and after MDT discussion, lymph node metastasis and lymphoma were excluded, and a final diagnosis of cervical LNTB was made.

##### 2.3.2.1 Final comprehensive diagnoses

(1) Therapy-related acute myeloid leukemia (t-AML), positive for *KMT2A-PTD* fusion gene, with mutations in *BRCA2*, *DNMT3A* and *IDH2* genes, high-risk group; (2) Cervical lymph node tuberculosis; (3) Squamous cell carcinoma of the right upper lobe (ypT0N0M0, Stage I).

### 2.4 Current treatment strategy and preliminary efficacy

Based on the patient’s clinical condition, age and physical status, an individualized comprehensive treatment strategy was formulated following multidisciplinary team (MDT) discussion involving specialists from Hematology, Infectious Diseases, Pharmacy, Oncology, Respiratory Medicine and Radiology. Considering the stable status of the pulmonary malignancy, administration of pembrolizumab was suspended. The core therapeutic principle was concomitant anti-tuberculosis and anti-leukemia therapy combined with supportive care, avoidance of drug-drug interactions, and regular re-evaluation after treatment. The detailed regimen is as follows:

#### 2.4.1 Anti-t-AML therapy

After two weeks of anti-tuberculosis treatment, the patient’s persistent low-grade fever resolved, and inflammatory markers (CRP, ESR) decreased. In accordance with the patient’s underlying diseases and laboratory findings, induction chemotherapy for t-AML was administered using the VA regimen. Previous studies have demonstrated that the combination of venetoclax and azacitidine synergistically induces apoptosis and activates mitochondrial apoptotic pathways in AML cells, downregulates Myeloid cell leukemia-1 (*MCL-1)* expression, and improves treatment tolerance (21). The VA regimen consisted of venetoclax 400 mg orally once daily (qd) plus azacitidine 75 mg/m^2^ subcutaneously on days (1–7). Concomitant supportive and symptomatic treatments were provided, including fluid supplementation, antiemetic therapy, acid suppression for gastric mucosal protection, and maintenance of electrolyte balance. Given the positivity of the *KMT2A-PTD* fusion gene, the menin inhibitor revumenib 270 mg orally twice daily (bid) was added in combination.

#### 2.4.2 Anti-tuberculosis therapy

The patient received induction chemotherapy with the VA regimen for t-AML. Venetoclax is primarily metabolized by cytochrome P450 3A4 (*CYP3A4*). Rifampicin and rifapentine are potent *CYP3A4* inducers that can markedly reduce the plasma concentration of venetoclax and thus compromise the efficacy of anti-t-AML treatment. Therefore, a modified quadruple anti-tuberculosis regimen was adopted for this patient, following the principles of early, combined, appropriate-dose, regular and full-course treatment.

The regimen included: moxifloxacin 400 mg orally qd, isoniazid 300 mg orally qd, pyrazinamide 500 mg orally three times daily (tid), and ethambutol 750 mg orally qd, with a planned treatment duration of 12 months. Hepatic function and tuberculosis-related markers (ESR, CRP) were monitored regularly during treatment, and drug dosages were adjusted according to adverse reactions.

**Figure 10:**
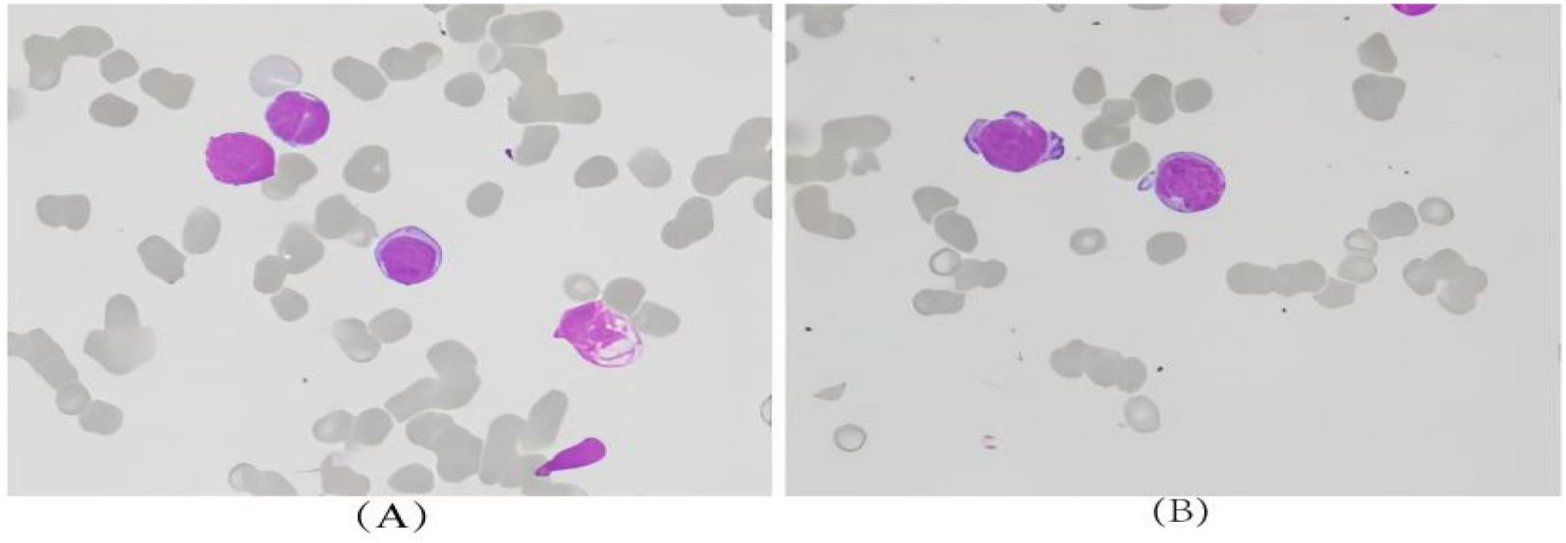
**(A, B)** Bone marrow aspirate findings: The specimen was adequately obtained, well-smeared and well-stained, with positive particles and fat droplets. The marrow was hypocellular, with a myeloid-to-erythroid (M/E) ratio of 4.29 (G = 53%, E = 17%). The myeloid lineage was normal in proportion but with an increased percentage of myeloblasts. The erythroid lineage was normal in proportion, dominated by intermediate and late erythroblasts, and mature erythrocytes showed essentially normal morphology. The lymphoid lineage was normal and consisted of mature lymphocytes. A total of 32 megakaryocytes were observed in the smear, with scattered platelets.

Two weeks after induction chemotherapy, follow-up tests showed bone marrow myeloblasts decreased to 32%, indicating partial remission (PR) (**Figure 10**). Routine blood tests revealed WBC 1.21×10^9^/L, RBC 1.69×10^12^/L, Hb 53 g/L, PLT 189×10^9^/L, ANC 0.69×10^9^/L, neutrophil percentage 56.5%, and CRP 22.99 mg/L. The patient’s low-grade fever and fatigue improved. Currently, the patient continues consolidation therapy for tuberculosis and leukemia with regular follow-up, without uncontrollable severe adverse events.

## 3 Discussion

### 3.1 Core characteristics of the case and analysis of its rarity

A middle-aged male patient with locally advanced lung squamous cell carcinoma (LSCC) underwent video-assisted thoracoscopic lesion resection after neoadjuvant therapy with (carboplatin + nab-paclitaxel + pembrolizumab), followed by postoperative maintenance therapy with pembrolizumab monotherapy; shortly thereafter, he developed secondary therapy-related acute myeloid leukemia (t-AML) and lymph node tuberculosis (LNTB) concurrently.

The core clinical features and exceptional rarity of this case are underscored by two key observations. First, the temporal presentation is distinctive: two life−threatening disorders developed concurrently shortly after completion of comprehensive therapy for LSCC, particularly during the postoperative immune maintenance phase, deviating from the typical clinical pattern of single complications. Second, this represents an unprecedented disease combination: to the best of our knowledge, no prior reports in the literature describe concurrent secondary t−AML and LNTB in patients with LSCC treated with neoadjuvant chemoimmunotherapy, surgical resection, and postoperative single−agent immune maintenance. Most existing studies document only one of these two complications in isolation (5, 22). Importantly, while t-AML is a well-recognized complication of cytotoxic chemotherapy and radiotherapy, and therapy-related myeloid neoplasms are widely reported in the literature—with DNA-damaging agents such as platinum compounds and topoisomerase II inhibitors known to drive genomic instability and clonal evolution in hematopoietic stem and progenitor cells, leading to secondary hematologic malignancies—lymph node tuberculosis also represents a relatively common form of extrapulmonary tuberculosis in immunocompromised hosts (23–25). By contrast, the synchronous occurrence of both conditions in this clinical setting is extremely rare and markedly amplifies the challenges of clinical diagnosis and management.

### 3.2 Pathogenetic mechanisms and recent research advances of t-AML secondary to LSCC following neoadjuvant therapy, surgical resection, and postoperative ICIs monotherapy maintenance

T-AML is a rare but severe long−term complication following comprehensive anticancer treatments including radiotherapy, chemotherapy and immunotherapy for malignant tumors. Its pathogenesis is complex and remains incompletely elucidated. It is critical to establish the hierarchical contribution of different therapies to t-AML development: cytotoxic chemotherapy represents the well-documented primary driver of t-AML via DNA damage and clonal selection in hematopoietic stem/progenitor cells, whereas the potential role of immune checkpoint inhibitors remains largely speculative and lacks robust clinical validation. Based on the treatment course of the present patient and recent up−to−date literature, the development of t-AML in this case is most likely attributable to the following factors:

#### 3.2.1 Cytotoxic effects of chemotherapeutic agents

Chemotherapy and/or radiotherapy can selectively induce the expansion of mutant clones with higher resistance to DNA damage in the bone marrow hematopoietic stem cell (HSC) and/or progenitor cell populations. Following exposure to chemotherapy and/or radiotherapy, the normal hematopoietic stem cell pool is damaged and depleted, whereas mutant clones proliferate further due to their survival advantage. Cytotoxic therapy targeting stem cells can trigger DNA damage, manifested as secondary mutations and/or chromosomal abnormalities, which in turn promotes the full formation of leukemic clones and leads to alterations in the bone marrow microenvironment. This process involves bone marrow stromal cells and T-cell subsets, including cytotoxic T lymphocytes and regulatory T cells (26, 27). Notably, the relatively short 4−month interval from the initiation of maintenance immunotherapy to t−AML onset does not preclude a diagnosis of therapy−related myeloid neoplasm. Short−latency t−AML, occurring within months to 1–2 years after exposure, has been well documented following platinum−based chemotherapy, and likely reflects rapid clonal expansion of chemotherapy−damaged hematopoietic stem and progenitor cells (5).

#### 3.2.2 Effects associated with immune checkpoint inhibitors

Immune-related adverse events (irAEs) affect multiple organs, predominantly the gastrointestinal tract, endocrine glands, skin and liver (28). In patients with advanced non-small cell lung cancer (NSCLC), the most frequent irAEs during pembrolizumab treatment are hypothyroidism, hyperthyroidism and pneumonitis (29), while hematologic toxicities include anemia, thrombocytopenia and platelet factor-associated acquired hemorrhagic disorders (30). To date, no definitive studies have established a causal link between irAEs and therapy-related acute myeloid leukemia (tAML). In the present case, the patient was diagnosed with tAML after 5 cycles of postoperative maintenance therapy with pembrolizumab monotherapy, and the contributory role of prior chemotherapeutic drugs in its pathogenesis could not be excluded. Furthermore, immunotherapy has been associated with the development of hyperprogressive disease (HPD) (31), and ICIs can also induce both HPD and pseudoprogression (32). In this patient, the clinical presentation was confirmed as t-AML and lymph node tuberculosis (LNTB), which excluded the possibility of ICI-related pseudoprogression or HPD. Notably, clinical reports of ICI-induced acute myeloid leukemia are extremely rare, and the correlation between these two entities remains controversial. Meanwhile, ICIs can disrupt immune tolerance and elevate the risk of *Mycobacterium tuberculosis* reactivation and dissemination (15, 16). In this patient, maintenance ICI therapy may have further compromised immune control over latent TB, thereby contributing to the development of lymph node tuberculosis.

#### 3.2.3 Gene mutations

Clonal hematopoiesis of indeterminate potential (CHIP) represents a key pre-existing risk factor for t-AML (33, 34). Cytotoxic chemotherapy promotes clonal expansion of CHIP-related mutations (*DNMT3A, IDH2*), leading to early-onset t-AML (35). The short latency does not support incidental *de novo* AML. The diagnosis of t-AML is supported by: (1) prior exposure to platinum and taxane chemotherapy; (2) typical t-AML-associated mutations (*KMT2A-PTD, DNMT3A, IDH2*); (3) no prior hematologic disorders. The *BRCA2* variant allele frequency (VAF) is ~48%, consistent with a heterozygous state. Germline testing was not performed, so a germline contribution cannot be confirmed (36). This alteration is most likely somatic in the setting of therapy-related myeloid neoplasm.

The most common gene mutations in AML include mutations in FMS-like tyrosine kinase 3(*FLT3*), nucleophosmin 1 (*NPM1*), *IDH2*, *DNMT3A*, *TP53*, neuroblastoma RAS viral oncogene homolog (*NRAS*) and complex mutations (37). Clinical studies indicate that AML patients harboring comutations usually have a poor prognosis (38). In the present patient, mutations in *DNMT3A*, *BRCA2* and *IDH2* were simultaneously detected; this mutational combination has not been reported in the literature, representing a rare case. Previous studies have reported that patients harboring concurrent *IDH2* and *DNMT3A* mutations have an unfavorable prognosis (37, 38). As the present patient carried both mutations, its therapeutic efficacy and prognosis warrant evaluation during subsequent treatment and follow-up. Furthermore, small-molecule targeted drugs against *DNMT3A-*mutated AML have been gradually developed in recent years, but their actual clinical efficacy still needs to be further verified (21). Current targeted therapies for AML are mainly indicated for patients with *FLT3* or *IDH2* mutations. The present patient harbored only an *IDH2* mutation, with no *FLT3* mutation. Studies have shown that enasidenib reduces the variant allele frequency of *IDH2* and improves molecular clearance, which is closely associated with the achievement of complete remission in patients. Meanwhile, this drug can induce a therapeutic response in approximately 40% of patients with *IDH2*-mutated AML, and the response rate is not affected by previous treatment responses (39), providing a new strategy for the subsequent treatment of this patient.In addition, the present patient was positive for the *KMT2A-PTD* fusion gene. Identification of specific KMT2A rearrangements (*KMT2A−r*) not only affects risk stratification in AML patients, but also has important clinical significance for disease monitoring and treatment decision−making (40). Studies have found that *KMT2A-PTD* mutations are present in 6% of patients with *de novo* AML and are associated with adverse outcomes (41), further emphasizing the necessity of identifying this mutation in the clinical management of AML patients. This importance is increasingly prominent especially with the gradual emergence of novel targeted therapies against *KMT2A-r* such as menin inhibitors (42). Given the positivity of *KMT2A-PTD*, the patient received oral revumenib in combination with the VA regimen. Subsequent re-examination showed improvement in hematological parameters compared with baseline, confirming the safety and efficacy of this combined regimen. Continuous observation and follow-up are still required.

### 3.3 Pathogenetic mechanisms underlying LNTB comprehensive treatment for LSCC

LNTB is a common form of extrapulmonary tuberculosis, with Mycobacterium tuberculosis infection as its primary etiology and immunocompromise as a major predisposing factor. The development of LNTB in this patient after comprehensive treatment for LSCC was mainly related to the following factor;

Patients with LSCC inherently exhibit tumor-related immunosuppression, and chemotherapy and immunotherapy further impair cellular and humoral immunity (43). This results in a marked reduction in host resistance to Mycobacterium tuberculosis. The weakened immune system can neither effectively eliminate latent Mycobacterium tuberculosis nor prevent new infection, ultimately leading to LNTB. Studies have shown that the incidence of tuberculosis infection in patients with malignant tumors following chemotherapy and immunotherapy is significantly higher than in the general population, with LNTB being the most common manifestation (9, 43, 44), which is consistent with the clinical presentation of our patient.

### 3.4 Therapeutic strategy and drug-drug interaction considerations

When t-AML and LNTB occur concurrently, the treatment regimen must balance anti-tuberculosis and anti-leukemic therapies, avoid drug-drug interactions, and weigh therapeutic efficacy against adverse reactions, which represents a major focus and challenge in clinical practice. Based on the treatment experience of the present case and recent literature, the therapeutic principles and precautions for the coexistence of these two diseases are as follows:

#### 3.4.1 Therapeutic sequence

No unified consensus has been established regarding the optimal treatment sequence. One study (45) reported that in 13 patients with newly diagnosed acute leukemia complicated by tuberculosis, the first induction chemotherapy was delayed for 2 months until tuberculosis was controlled, after which leukemia remission was achieved. However, 2 patients with newly diagnosed AML and tuberculosis in that cohort failed to achieve remission after induction chemotherapy following a 2-month delay and eventually died. Another study (21) showed that in AML patients with tuberculosis, anti-leukemic combination chemotherapy was initiated after approximately 2 weeks of anti-tuberculosis therapy with the HRZE regimen and sustained negative sputum smears. Subsequent follow-up revealed persistently negative sputum smears and gradual resolution of tuberculosis cavities in both lungs on chest CT, confirming the safety and efficacy of this strategy. Therefore, patients with high-risk AML and active tuberculosis should receive timely anti-leukemic therapy on the basis of active anti-tuberculosis treatment to maximize clinical benefit.

In the present case, anti-leukemic therapy was initiated after approximately 2 weeks of anti-tuberculosis therapy with the HZEM regimen, when the patient’s fatigue and low-grade fever subsided. Subsequent bone marrow biopsy showed a marked reduction in the proportion of myeloblasts and improvement in related parameters, further validating the safety and efficacy of this regimen.

#### 3.4.2 Considerations of drug-drug interactions

Potential interactions exist between anti-tuberculosis drugs and anti-leukemic agents, which may reduce therapeutic efficacy or increase the risk of adverse reactions. Notably, rifampicin/rifapentine are potent *CYP3A4* inducers that can accelerate the metabolism of chemotherapeutic drugs such as taxanes and topoisomerase inhibitors, lower their plasma concentrations and weakening anti-leukemic effects. Therefore, moxifloxacin was used in place of rifampicin/rifapentine for anti-tuberculosis therapy in this patient. Close monitoring of plasma drug concentrations, as well as liver function, routine blood tests and other indicators, is required in clinical practice, with timely intervention for adverse reactions.

#### 3.4.3 Importance of supportive care

Patients with concurrent lung squamous cell carcinoma and tuberculosis present with profoundly impaired immune function and are highly susceptible to severe complications such as infection and hemorrhage. Supportive care serves as a critical guarantee for the smooth implementation of treatment. Adequate nutritional support and symptomatic treatment should be provided. Anemia and thrombocytopenia must be corrected promptly, and infection prophylaxis should be implemented. Meanwhile, enhanced nursing care is required to avoid exposure to cold and traumatic injury. Relevant laboratory indicators should be regularly re-evaluated, and the therapeutic regimen adjusted in a timely manner.

### 3.5 Clinical implications

Based on this patient’s diagnosis, treatment and the latest literature, the clinical implications are as follows: ① For locally advanced LSCC patients receiving comprehensive therapy (immunochemotherapy plus surgery and immune maintenance), strengthen long-term follow-up, particularly routine blood and bone marrow aspiration monitoring, to early detect hematological abnormalities and screen for severe complications like t-AML. ② Prioritize tuberculosis-related screening in patients with unexplained fever and lymphadenopathy post-comprehensive cancer treatment to avoid LNTB misdiagnosis; maintain vigilance for latent tuberculosis reactivation in immunocompromised individuals. Treatment plans should be formulated, with emphasis on drug interactions and supportive care, to improve treatment efficacy and patient prognosis. ③ For concurrent diseases, fully leverage MDT to integrate hematology, infectious diseases, oncology and other disciplinary resources, and formulate individualized treatment plans.

### 3.6 Importance of modern genomic diagnostics and personalized oncology

The comprehensive genomic profiling performed in this case underscores the pivotal role of modern molecular diagnostics in contemporary oncology and hematology. In the era of precision medicine, advanced genomic strategies—including whole-genome sequencing (WGS) (46), long-read sequencing platforms (e.g., PacBio, Oxford Nanopore) (47), and broad-based next-generation sequencing panels (48) —enable high-resolution detection of gene mutations, fusions, copy number alterations, and structural variants that are not readily identified by conventional cytogenetics or targeted assays. Such approaches allow for earlier and more precise identification of actionable genomic alterations, including the *IDH2* mutation and *KMT2A*-PTD fusion detected in this patient, which directly guided the use of targeted agents such as the menin inhibitor revumenib in addition to conventional chemotherapy (42). By moving beyond traditional cytotoxic chemotherapy alone, these diagnostic advances facilitate truly personalized therapeutic regimens that improve efficacy, minimize toxicities, and refine prognostic stratification (49). In the context of therapy-related myeloid neoplasms and concurrent complications such as tuberculosis, comprehensive genomic characterization is particularly valuable for balancing competing risks, avoiding drug–drug interactions, and tailoring treatment to the individual’s molecular and clinical profile. Broader implementation of these modern genomic tools will continue to enhance early detection, mechanistic understanding, and personalized management of rare treatment-related complications, thereby strengthening the translational impact of clinical case-based research (50).

## 4 Conclusions

T-AML and LNTB following treatment for LSCC represent clinically challenging complications. These events illustrate the dual pathophysiological risks of modern anticancer therapy: treatment-related genotoxicity and immunosuppression.

Platinum-based regimens and topoisomerase II inhibitors induce DNA damage and clonal evolution in hematopoietic stem/progenitor cells, predisposed to t-AML. Meanwhile, treatment-related lymphopenia and impaired cellular immunity facilitate reactivation or *de novo* infection of Mycobacterium tuberculosis, leading to LNTB. As interrelated complications, they highlight the critical balance between therapeutic efficacy and toxicity.

This rare and life-threatening scenario requires multidisciplinary collaboration among oncologists, hematologists, and infectious disease specialists, indicating that long-term cancer management has entered an era of survivorship care dedicated to high-quality long-term survival.

## Data Availability

The original contributions presented in the study are included in the article/supplementary material. Further inquiries can be directed to the corresponding author.
